# An Insidious Case of Rapidly Progressive Glomerulonephritis Secondary to Pauci-Immune Crescentic Glomerulonephritis

**DOI:** 10.7759/cureus.73233

**Published:** 2024-11-07

**Authors:** Vasilis C Mavratsas, Lan Vu, Owen L Yeh

**Affiliations:** 1 Internal Medicine, University of Texas Medical Branch, Galveston, USA

**Keywords:** anca-associated vasculitis, constitutional symptoms, delay in diagnosis, pauci-immune crescenting glomerulonephritis, rapidly progressive glomerulonephritis (rpgn)

## Abstract

Rapidly progressive glomerulonephritis (RPGN) is a severe type of nephritic syndrome that involves progressive loss of kidney function and can lead to significant morbidity and mortality. RPGN has many etiologies, of which pauci-immune crescenting glomerulonephritis (PICGN) is the most common; however, patients often present with nonspecific symptoms, which can lead to a delay in diagnosis and treatment. We describe one such case that was correctly identified only after multiple clinic and hospital encounters. Following the correct diagnosis, the patient was started on induction therapy with corticosteroids and rituximab with plans for outpatient maintenance therapy. We have included a discussion of the pathogenesis, epidemiology, diagnosis and treatment of RPGN, highlighting the importance of early recognition of the disease in achieving better patient outcomes.

## Introduction

Glomerulonephritis refers to a diverse group of diseases that cause inflammation of the glomeruli, which are the filtration units of the kidney [1,2]. Since it involves direct damage to the microscopic architecture of the kidney, it is a type of intra-renal injury, and the gold-standard diagnostic test is a kidney biopsy [3,4]. Glomerulonephritis can vary greatly in terms of its severity, rate of progression, and degree of permanent injury [1]. More severe forms can manifest as nephritic syndrome, characterized by hematuria, proteinuria, elevated blood pressure, and edema, often in the setting of elevated serum creatinine [5]. Glomerulonephritis is estimated to be the cause of 15% of cases of end-stage renal disease (ESRD) in the United States [6]. A diagnosis of glomerulonephritis in the Medicare population has been associated with a 2.7-fold to 3.9-fold increase in mortality rate per 1000 patient-years [7].

As the name suggests, rapidly progressive glomerulonephritis (RPGN) is a severe type of glomerulonephritis with multiple possible etiologies that involves progressive loss of kidney function over a period of days to months and can lead to ESRD and an overall poor prognosis, especially if left untreated [1,4,8,9]. Pauci-immune crescentic glomerulonephritis (PICGN) is one of the causes of RPGN, yet despite recent advancements in the understanding and management of the disease, PICGN can present with nonspecific symptoms that result in delayed diagnosis and treatment, ultimately leading to worse patient outcomes [3-5,8,10]. In this paper, we present an insidious case of RPGN caused by PICGN.

## Case presentation

The patient was a 72-year-old White Hispanic male with no known significant past medical history before the events described herein. Four months prior to hospitalization, the patient presented to his primary care physician, reporting a sensation of “tiredness” or fatigue in his legs. Initial lab work was unremarkable, and he was referred to podiatry. Evaluation yielded no significant findings at the time. Over the next month, the patient’s symptoms extended to his hips and shoulders, and he also developed intense pain in his calves, joints, and groin. He started requiring a walker to ambulate and, even with the walker, could not ambulate for more than 15 minutes at a time. He was then referred to rheumatology, where he was diagnosed with polymyalgia rheumatica and started on a short course of high-dose oral corticosteroids. His symptoms improved within five days of treatment but then quickly relapsed following cessation of therapy. At that time, the patient also developed urinary incontinence and increased urinary frequency. He was then started on methotrexate, but it was discontinued due to severe anemia. Soon thereafter, he presented to the emergency department (ED) for chest tightness and diarrhea. He was found to be COVID-19 positive and had an elevated troponin, but the subsequent cardiac workup was unremarkable, including a transthoracic echocardiogram and cardiac catheterization.

Following the initial ED visit, the patient was seen by a hematologist to evaluate his anemia and was told to go to the ED once again for a blood transfusion. There, he received one unit of packed red blood cells (pRBCs) and was subsequently hospitalized for acute kidney injury seen on basic lab work (Table 1). Urinalysis was significant for hematuria and pyuria (Table 1).

**Table 1:** Basic laboratory data Measurements are from blood samples unless otherwise specified. WBC, white blood cells; RBC, red blood cells; MCV, mean corpuscular volume; MCHC, mean corpuscular hemoglobin concentration; RDW - SD, red cell distribution width - standard deviation; TIBC, total iron-binding capacity; BUN, blood urea nitrogen; eGFR, estimated glomerular filtration rate; LE, leukocyte esterase; HPF, high-power field

Parameter	Patient values	Reference values
WBC (x10^3/uL)	10.21	4.2 - 10.7
RBC (x10^6/uL)	3.13	4.26 - 5.52
Hemoglobin (g/dL)	8.2	12.2 - 16.4
Hematocrit (%)	26.4	38.4 - 49.3
MCV (fL)	84.3	81.7 - 95.6
MCHC (g/dL)	31.1	31.2 - 35.0
RDW - SD (fL)	54.1	38.5 - 51.6
Platelets (x10^3/uL)	299	150 - 328
Iron (ug/dL)	74	50 - 160
Ferritin (ng/mL)	663	18 - 464
TIBC (ug/dL)	173	250 - 410
Transferrin saturation (%)	43	20 - 50
Sodium (mmol/L)	133	135 - 145
Potassium (mmol/L)	4.7	3.5 - 5.0
Chloride (mmol/L)	103	98 - 108
CO2 Total (mmol/L)	18	23 - 31
Anion Gap	12	2 - 16
BUN (mg/dL)	92	7 - 23
Glucose (mg/dL)	87	70 - 110
Creatinine (mg/dL)	5.76	0.60 - 1.25
eGFR (mL/min/1.73m^2)	9.7	>90
Calcium (mg/dL)	8.2	8.6 - 10.6
Phosphorus (mg/dL)	4.3	2.5 - 5.0
Magnesium (mg/dL)	2.2	1.7 - 2.4
Color Urine	Amber	Yellow
Appearance Urine	Cloudy	Clear
Specific Gravity Urine	1.032	1.003 - 1.030
pH Urine	5	4.8 - 8.0
Protein Urine (mg/dL)	100	Negative
Glucose Urine (mg/dL)	50	Negative
Ketones Urine	Negative	Negative
Bilirubin Urine	Negative	Negative
Blood Urine	2+	Negative
Urobilin Urine	Normal	Normal
Nitrite Urine	Negative	Negative
LE Urine (leukocytes/uL)	500	Negative
RBC/HPF Urine	42	0 - 3
WBC/HPF Urine	> 182	0 - 5

Subsequent studies were significant for a positive antinuclear antibody (ANA) and myeloperoxidase (MPO) antineutrophil cytoplasmic antibody (ANCA), negative proteinase 3 (PR3) ANCA, normal C3 and C4 complement protein levels, and negative hepatitis B and C serologies (Table 2).

**Table 2:** Autoimmune workup and serologies ANA, antinuclear antibody; Ab, antibody; GBM, glomerular basement membrane; IgG, immunoglobulin G; IgM, immunoglobulin M; HIV, human immunodeficiency virus

Parameter	Patient values	Reference values
ANA	Positive	Negative
ANA Titer	1:40	≤ 1:40
C3 Complement (mg/dL)	86	86 - 184
C4 Complement (mg/dL)	26	20 - 59
Proteinase-3 Ab (U/mL)	< 0.6	≤ 2.0
Myeloperoxidase Ab (U/mL)	41	≤ 3.5
GBM IgG (AU/mL)	0	0 - 19
Hepatitis B Surface Ab	Negative	Negative
Hepatitis B Surface Antigen	Negative	Negative
Hepatitis B Core Ab IgM	Negative	Negative
Hepatitis C Ab	Negative	Negative
HIV 1/2 Antigen-Ab	Negative	Negative

**Figure 1:**
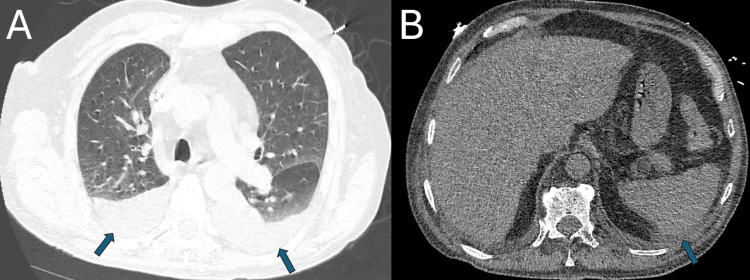
CT imaging without contrast: (A) Moderate bilateral layering pleural effusions (indicated by the blue arrows) and trace interstitial edema. (B) Mild wall thickening and haziness of the peripheral fat, and collection of fluid around the spleen, suggestive of perisplenic ascites (indicated by the blue arrow).

He required hemodialysis and underwent a kidney biopsy that revealed PICGN and necrotizing arteritis. The patient became febrile and anuric on his second day of hemodialysis. He received one additional unit of pRBCs and was started on pulse-dose intravenous corticosteroids. Computed tomography (CT) imaging was consistent with anasarca (Figures 1A, 1B).

The patient was subsequently transferred to another hospital that offered inpatient rheumatology services. There, he completed the course of pulse dose corticosteroids. Rheumatology confirmed the diagnosis of PICGN and started rituximab and high-dose oral prednisone daily for two weeks with subsequent tapering. He continued to be dialysis-dependent throughout his hospitalization. Prior to discharge, a tunneled hemodialysis catheter was placed, and follow-up appointments were scheduled with rheumatology and nephrology, with a tentative plan to start treatment with azathioprine and avacopan as an outpatient.

## Discussion

The etiologies of RPGN are broadly separated into three major categories depending on the histopathological patterns of immune complex deposition [8,9,11]. The most common category that causes RPGN is PICGN, which refers to the paucity or absence of immune complex deposition as compared to the other two categories [9,11,12]. PICGN is usually associated with ANCA, with the term PICGN referring to the renal manifestation of ANCA-associated vasculitis (AAV), although PICGN can rarely present without ANCA but with otherwise similar features to ANCA-associated PICGN [12,13]. The second category involves linear depositions of anti-glomerular basement membrane (anti-GBM) antibodies, often referred to as Goodpasture syndrome [8,11,14]. The third category consists of any disease that can cause immune complex deposition in the glomeruli, such as systemic lupus erythematosus or IgA nephropathy [8,11].

The incidence of RPGN in the United States has been reported to be around seven cases per million people per year [15]. RPGN involves a rapid loss of kidney function over a period of days to months, indicated by a decrease in urine output, a decrease in glomerular filtration rate, and an increase in serum creatinine, with early symptoms often being nonspecific, such as edema, shortness of breath, fever, and fatigue [8,11]. Systemic etiologies of RPGN can present with extrarenal signs and symptoms, such as hemoptysis, epistaxis, and melena [8,11]. Urinalysis will be significant for hematuria and dysmorphic red blood cells, as well as proteinuria [8,11]. Other laboratory testing will often show anemia, leukocytosis, and elevated inflammatory markers [8,11]. Further laboratory and histopathologic testing results will depend on the particular etiology of each case of RPGN [8,11]. Among the aforementioned etiologies of RPGN, PICGN is the most common cause, comprising 40% to 50% of cases, while immune complex deposition is responsible for 25% to 30%, with the remainder attributed to anti-GBM disease [8]. Of note, there is a paucity of recent epidemiological studies on RPGN in isolation as well as in relation to its various causes, which warrants further research.

The diagnosis of PICGN is achieved through the integration of clinical, serologic, radiologic, and histopathologic data, usually with the help of a rheumatologist [16]. Histopathologic data, such as the abundance of cellular crescents, can be suggestive of the likelihood of renal recovery [17]. Several classification criteria have been developed over the past few decades, with advancements in the understanding of pathogenesis and improvement of diagnostic tools leading to the refinement of those guidelines over time [18]. The presence of ANCA is responsible for the pathogenesis of PICGN, as ANCA binds to and activates neutrophils, which causes them to initiate inflammatory cascades, such as the alternative complement pathway, that damage intrarenal self-antigens located on structures such as podocytes or the glomerular basement membrane, ultimately leading to glomerular damage and impaired kidney function [2,19].

The treatment of ANCA-associated vasculitis can be separated into induction and maintenance phases [12,20]. The induction phase involves the use of corticosteroids in combination with cyclophosphamide, rituximab, or avacopan [12,20,21]. Cyclophosphamide is a DNA-alkylating agent with antineoplastic and immunosuppressive properties [22]. It has been shown to influence the function of regulatory T cells and cause depletion of B cells [20,22]. Rituximab is a monoclonal antibody that targets CD20 on B cells [16]. Avacopan is a C5a receptor antagonist that ultimately inhibits neutrophil activation [21]. Other options for induction include plasma exchange and methotrexate [12,20]. The mechanism of action of methotrexate in its role as an immunosuppressant is complex and multifaceted but partly involves the reduction of proliferation of various immune system cells by blocking folate-dependent enzymatic steps required in the production of nitrogenous bases that are needed in the synthesis of DNA and RNA [23]. Rituximab is considered the first-line treatment for the maintenance of remission, with azathioprine being an alternative choice when rituximab is contraindicated [12,16]. Azathioprine inhibits purine synthesis and blocks DNA replication through the incorporation of its active metabolites into DNA strands [24].

## Conclusions

Constitutional or nonspecific symptoms can, by definition, be associated with an array of disease processes, ranging from acute to chronic, from benign to malignant, and from self-resolving to those that require timely disease-modifying therapy. It is, therefore, expected that patients who present with constitutional symptoms may experience a delay in diagnosis or, as in the case presented here, be misdiagnosed until the progression of the disease necessitates a more thorough and ultimately successful workup. Although it is understandable to first consider more common conditions in the differential diagnosis, consideration of rarer and more severe diseases is also important, particularly if their course can be altered with prompt treatment. Cases of PICGN leading to RPGN comprise one such category of relatively rare, commonly misdiagnosed, but potentially treatable diseases.

The evaluation of the patient by multiple different healthcare providers at various healthcare institutions was an additional barrier to optimal care, as each encounter involved an incomplete transfer of information from prior encounters that led to redundancy in testing and evaluation of the patient, but also made the case susceptible to biases such as anchoring bias or confirmation bias. Coordination between multiple specialty and subspecialty teams, while helpful and often necessary, can also increase complexity due to delays in communication, errors stemming from miscommunication, or even a misalignment of opinions. Epidemiologic studies that raise awareness and improve the ability to quickly recognize and treat RPGN should improve patient outcomes.
